# Is dietary intake of antioxidant vitamins associated with reduced adverse effects of air pollution on diabetes? Findings from a large cohort study

**DOI:** 10.1016/j.ecoenv.2022.114182

**Published:** 2022-11

**Authors:** Haopeng Li, Miao Cai, Haitao Li, Zhengmin (Min) Qian, Katie Stamatakis, Stephen Edward McMillin, Zilong Zhang, Qiansheng Hu, Hualiang Lin

**Affiliations:** a School of Public Health, Sun Yat-sen University, Guangzhou 510080, Guangdong, China; b Department of Social Medicine and Health Service Management, Health Science Center, Shenzhen University, Shenzhen 518060, Guangdong, China; c Department of Epidemiology and Biostatistics, College for Public Health and Social Justice, Saint Louis University, St. Louis, MO 63104, USA; d School of Social Work, College for Public Health and Social Justice, Saint Louis University, St. Louis, MO 63103, USA

**Keywords:** Diabetes mellitus, Vitamin A, Vitamin C, Vitamin E

## Abstract

**Introduction:**

It remains unknown whether higher dietary intake of antioxidant vitamins could reduce the harmful effects of air pollution on incident diabetes mellitus.

**Methods:**

A total of 156,490 participants free of diabetes mellitus in the UK Biobank data were included in this analysis. Antioxidant vitamin intake was measured using a 24-h food intake questionnaire, and results were categorized as sufficient or insufficient according to the British Recommended Nutrient Intake. Exposure to fine particles (PM_2.5_), thoracic particles (PM_10_), nitrogen dioxide (NO_2_), and nitrogen oxide (NO_x_) was estimated using land use regression models at participants’ residences. Incident diabetes mellitus was identified using health administrative datasets. Cox regression models were used to assess the associations.

**Results:**

A total of 4271 incident diabetes mellitus cases were identified during a median follow-up of 11.7 years. Compared with participants with insufficient intake of antioxidant vitamins, those with sufficient consumption had a weaker association between air pollution (PM_2.5_, PM_10_ and NO_2_) and diabetes mellitus [sufficient vs. insufficient: HR = 1.12 (95 % CI: 0.87, 1.45) vs. 1.69 (95 % CI: 1.42, 2.02) for PM_2.5_, 1.00 (95 % CI: 0.88, 1.14) vs. 1.21 (95 % CI: 1.10, 1.34) for PM_10_, and 1.01 (95 % CI: 0.98, 1.04) vs. 1.05 (95 % CI: 1.03, 1.07) for NO_2_ (all *p* for comparison < 0.05)]. Among different antioxidant vitamins, we observed stronger effects for vitamin C and E.

**Conclusion:**

Our study suggests that ambient air pollution is one important risk factor of diabetes mellitus, and sufficient intake of antioxidant vitamins may reduce such adverse effects of air pollution on diabetes mellitus.

## Highlights

• Long-term air pollution is associated with an increased risk of diabetes mellitus.
• Oxidative stress is an important mechanism underlying air pollution health effects.
• Sufficient dietary vitamins may reduce risks of diabetes related to air pollution.
• Vitamins C and E shows stronger effects among different antioxidant vitamins.

## 1 Introduction

Diabetes mellitus is one of the major causes of global burden of disease (Georgakis et al., 2021). It is projected that the global adult with diabetes mellitus will increase from 536.6 million in 2021 to 783.2 million by 2045 (Li et al., 2021, Sun et al., 2022). A few studies have suggested that long-term air pollution exposure was associated with an increased risk of diabetes mellitus (Yang et al., 2020, Zhang et al., 2021). For example, one meta-analysis synthesized 30 studies investigating the effects of ambient air pollutants on diabetes mellitus, and pointed out that it is necessary to find way to reduce the remarkable burden of diabetes mellitus attributable to air pollution exposure (Liu et al., 2019).

Oxidative stress has been proved to be one important biological mechanism underlying the air pollution health effects in both experimental and epidemiology studies (Gangwar et al., 2020, Ghio et al., 2012). The interaction of cells with air pollutants could directly produce pro-oxidants and activate redox sensitive pathways (Franklin et al., 2015). Meanwhile, this response could release a series of mediators into the systemic circulation, resulting in lipid peroxidation, activation of pro-inflammatory signaling, and depletion of antioxidants, inducing systemic effects including metabolic dysfunction (Lim and Thurston, 2019). Insulin resistance is related to the development of metabolic dysfunction, which ultimately contributes to diabetes mellitus and some other adverse health conditions (Lim and Thurston, 2019). Specifically, chronic exposure to PM_2.5_ could induce endothelial function impairment by increasing endothelial cell apoptosis through oxidative stress, which remained a suggested biological pathway linking air pollution to diabetes mellitus (Gorini et al., 2021). These findings have provided a biological plausibility for applying dietary interventions with antioxidant effects to reduce the health effects of air pollution.

Strengthening the body’s antioxidant capacity might be helpful to reduce the adverse health effects caused by air pollution exposure (Lin et al., 2018). Vitamins A, C and E are important antioxidants, which could reduce the body’s susceptibility to oxidative damage (Ghezzi, 2020). Previous studies suggested that vitamins A, C and E might have the potential to mitigate the harmful effects of air pollution on a few health outcomes, particularly those of the respiratory system. For example, one study reported that nitrogen oxide (NO_x_) from both endogenous and environmental sources might contribute to oxidative injury and asthma, and sufficient intake of vitamin C was protective against this adverse effect (Hatch, 1995). Some studies also suggested that high dietary intakes of beta-carotene, vitamins C and E could significantly reduce the risk of peak expiratory flow decrements caused by air pollution, especially by thoracic particles (PM_10_) (Grievink et al., 1999, Su et al., 2013). In general, these studies suggested that it seems reasonable to investigate the modification effect of dietary intake of antioxidant vitamins on air pollution-related health effects, and the results are easier to translated into practical recommendations for the public.

However, it remains largely unknown whether dietary intake of antioxidants could also modify the relationship between air pollution exposure and the risk of other health outcomes such as diabetes mellitus. We thus conducted this study with the aim to explore whether dietary intake of antioxidant nutrients (i.e., vitamin A, C and E) could attenuate the health effects of ambient air pollution exposure on the risk of diabetes mellitus.

## 2 Material and methods

### 2.1 Study population

We used longitudinal data from the UK Biobank, a prospective cohort study of 502,461 adults aged 37–73 years in the United Kingdom (UK). The participants were recruited from 22 assessment centers in the UK during 2006–2010. Participants were asked to complete a series of questionnaires and brief interviews, measurements of various physical indicators and collection of biomedical samples. The implementation of the UK Biobank was approved by the North West Multi-Center Research Ethics Committee (REC reference: 21/NW/0157) (Said et al., 2018), and all participants were asked to complete the electronic version of informed consent.

**Fig. 1:**
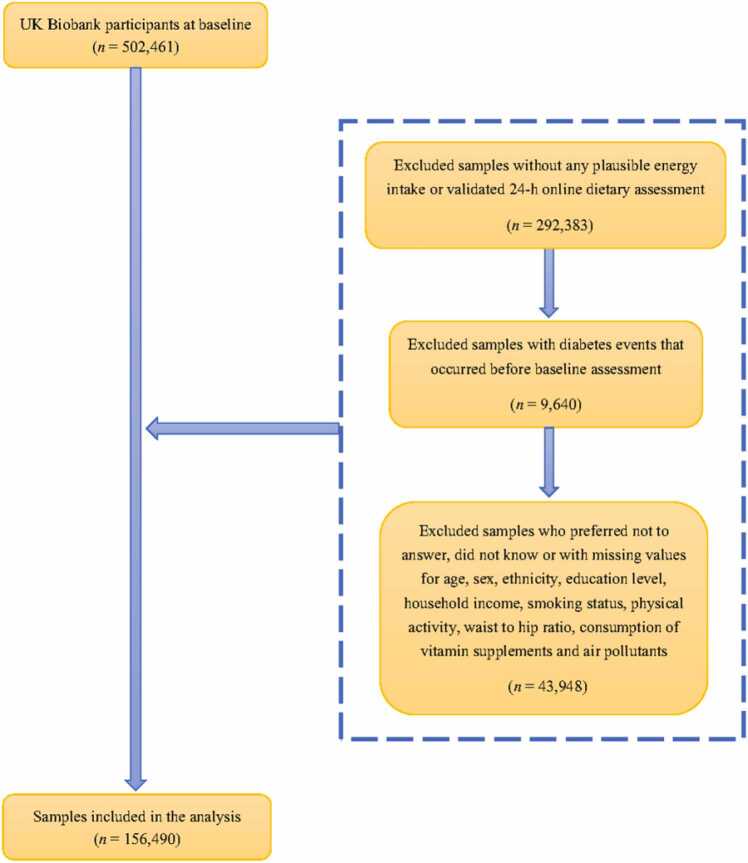
Flow chart of inclusion and exclusion of the study participants.

The process of participant selection in the present study is shown in Fig. 1. Among the 502,461 participants initially recruited, 292,383 were excluded as they did not provide at least one valid Oxford WebQ (24-h dietary recall web-based questionnaire) response or responses included implausible energy intakes. We further excluded 43,948 participants with missing values on important covariates, and 9640 with pre-existing diabetes mellitus based on baseline glycated hemoglobin, medical records, self-reported history of diabetes mellitus, or insulin medication use before baseline assessment, resulting in 156,490 participants included in data analysis.

### 2.2 Air pollution exposures

The UK Biobank used Land Use Regression (LUR) models to estimate the annual concentrations of several pollutants, including particulate matter with an aerodynamic diameter ≤ 2.5 µm (PM_2.5_), particulate matter with an aerodynamic diameter of smaller than 10 µm (PM_10_), nitrogen dioxide (NO_2_) and NO_x_. The LUR model was developed by the European Study of Cohorts for Air Pollution Effects project and has been validated in previous studies (Aung et al., 2018, Wang et al., 2021). Due to the availability of data, we obtained only the average concentrations of all pollutants in 2010, the annual average concentrations of NO_2_ from 2005 to 2007 and the mean concentrations of PM_10_ in 2007. Therefore, the yearly mean concentrations of all available years were estimated for each participant based on their residential address and were used as a proxy of long-term exposure.

### 2.3 Dietary intake of antioxidant vitamins

A touch screen questionnaire was used at baseline survey to collect information on the habitual consumption frequency of major foods and food categories in the past year. The UK Biobank also used a web-based 24-h dietary assessment (the Oxford WebQ) to measure the average 24-h dietary intake in over 200,000 participants. The Oxford WebQ calculated nutrient intakes through multiplying the size of each food serving by the number of servings consumed, and then multiplying it by the quantity of each nutrient in each food serving according to the UK Nutrient Databank Food Composition Tables (Piernas et al., 2021). Multiple dietary assessments were performed to illustrate the seasonal changes in dietary intake and to provide the estimate of habitual intake at baseline and subsequent follow-up visits (2009–2012). The assessment of each dietary variable was reckoned by averaging all five measurements during the follow-up period. For participants who completed dietary assessments on one or more occasions, we calculated average intakes of vitamin A equivalent according to the international uniform calculation formula: 1 g vitamin A equivalent = 1 g retinol + (1/12) g β-carotene, vitamin C, vitamin E and some other dietary variables (see Table 1) across all dietary assessments. According to the Nutrition Reference Values from the British Adult recommended nutrient intakes (RNIs) of dietary intake of vitamin A equivalent (≥ 700 μg), vitamin C (≥ 40 g), and vitamin E (≥ 13 g for male and ≥ 11 g for female) (British Nutrition Foundation, 2016; European Food Safety Authority, 2019), a binary variable was computed: sufficient (meeting the RNI for 2 or more antioxidants) and insufficient (meeting the RNI for less than 2 antioxidants) (Das et al., 2021).

**Table 1:** Baseline characteristics of the study participants.

Variables		Overall (*n* = 156,490)	Non-cases (*n* = 152,219)	Incident Diabetes Cases (*n* = 4271)	*P*
Sex, *n* (%)					
	Female	84,933 (54.27)	83,224 (54.67)	1709 (40.01)	< 0.01
	Male	71,557 (45.73)	68,995 (45.33)	2562 (59.99)	
Age, mean (SD), years		55.78 (7.98)	55.71 (7.99)	58.37 (7.39)	< 0.01
Ethnicity, *n* (%)					
	White	150,281 (96.03)	146,314 (96.12)	3967 (92.88)	< 0.01
	Asian	2408 (1.54)	2270 (1.49)	138 (3.23)	
	Black	1782 (1.14)	1694 (1.11)	88 (2.06)	
	Mixed	958 (0.61)	932 (0.61)	26 (0.61)	
	Others^a^	1061 (0.68)	1009 (0.66)	52 (1.22)	
Education attainment, *n* (%)					
	Higher degree	93,586 (59.80)	91,433 (60.07)	2153 (50.41)	< 0.01
	Any school degree	44,214 (28.25)	42,988 (28.24)	1226 (28.71)	
	Vocational qualifications	7270 (4.65)	6994 (4.59)	276 (6.46)	
	Other	11,420 (7.30)	10,804 (7.10)	616 (14.42)	
Household income, *n* (%)					
	Less than 18,000	20,618 (13.18)	19,669 (12.92)	949 (22.22)	< 0.01
	18,000–30,999	33,832 (21.62)	32,773 (21.53)	1059 (24.80)	
	31,000–51,999	41,233 (26.35)	40,213 (26.42)	1020 (23.88)	
	52,000 to 100,000	36,590 (23.38)	35,915 (23.59)	675 (15.80)	
	Greater than 100,000	11,228 (7.17)	11,084 (7.28)	144 (3.37)	
	Unknown	12,989 (8.30)	12,565 (8.25)	424 (9.93)	
Waist to hip ratio, mean (SD)		8.62 (0.88)	8.60 (0.87)	9.30 (0.85)	< 0.01
Physical activity, *n* (%)					
	Low	28,193 (18.02)	27,106 (17.81)	1087 (25.45)	< 0.01
	Moderate	66,145 (42.27)	64,373 (42.29)	1772 (41.49)	
	High	62,152 (39.72)	60,740 (39.90)	1412 (33.06)	
Smoking status, *n* (%)					
	Never	89,139 (56.96)	87,165 (57.26)	1974 (46.22)	< 0.01
	Previous	55,384 (35.39)	53,537 (35.17)	1847 (43.25)	
	Current	11,967 (7.65)	11,517 (7.57)	450 (10.54)	
Alcohol intake, mean (SD), g		16.69 (21.31)	16.73 (21.27)	15.30 (22.64)	< 0.01
Vitamin supplement, *n* (%)					
	No	110,437 (70.57)	107,418 (70.57)	3019 (70.69)	0.88
	Yes	46,053 (29.43)	44,801 (29.43)	1252 (29.31)	
Protein intake, mean (SD), g		81.84 (24.39)	81.77 (24.31)	84.33 (27.09)	< 0.01
PUF intake, mean (SD), g^b^		14.29 (7.11)	14.28 (7.11)	14.44 (7.28)	0.14
Total sugar intake, mean (SD), g		120.21 (47.66)	120.19 (47.52)	121.21 (52.17)	0.17
Fiber intake, mean (SD), g		16.41 (6.50)	16.41 (6.49)	16.25 (7.02)	0.11
Antioxidant vitamins intake, *n* (%)					
	Insufficient	97,957 (62.60)	95,163 (62.52)	2794 (65.42)	< 0.01
	Sufficient	58,533 (37.40)	57,056 (37.48)	1477 (34.58)	
Vitamin A intake, *n* (%)					
	Insufficient	111,412 (71.19)	108,350 (71.18)	3062 (71.69)	0.48
	Sufficient	45,078 (28.81)	43,869 (28.82)	1209 (28.31)	
Vitamin C intake, *n* (%)					
	Insufficient	12,465 (7.97)	12,023 (7.90)	442 (10.35)	< 0.01
	Sufficient	144,025 (92.03)	140,196 (92.10)	3829 (89.65)	
Vitamin E intake, *n* (%)					
	Insufficient	122,073 (78.01)	118,575 (77.90)	3498 (81.90)	< 0.01
	Sufficient	34,417 (21.99)	33,644 (22.10)	773 (18.10)	
PM_2.5_, mean (SD), μg/m^3^^b^		9.90 (1.02)	9.90 (1.02)	10.01 (1.01)	< 0.01
PM_10_, mean (SD), μg/m^3^^b^		19.37 (2.05)	19.37 (2.05)	19.49 (2.00)	< 0.01
NO_2_, mean (SD), μg/m^3^^b^		29.38 (9.77)	29.36 (9.78)	30.03 (9.43)	< 0.01
NO_x_, mean (SD), μg/m^3^^b^		43.08 (15.39)	43.04 (15.37)	44.68 (16.12)	< 0.01

a "Others" means any ethnicity other than White, Black, Asian, or Mixed ethnicity.

b PUF is an abbreviation of polyunsaturated fat, PM_2.5_ is an abbreviation of fine particles, PM_10_ is an abbreviation of thoracic particles, NO_2_ is an abbreviation of nitrogen dioxide, NO_x_ is an abbreviation of nitrogen oxide.

### 2.4 Follow-up and outcome ascertainment

All the participants were followed up using health administrative datasets of hospital admission, primary health care, and self-reported health data linked to the UK Biobank. Incident diabetes mellitus was identified according to the International Classification of Diseases 10th Revision (E10–E14). Hospital admission data were obtained through health episode statistics (England and Wales) and Scottish morbidity records (Scotland) (http://content.digital.nhs.uk/services). The all-data source registry-based follow-up was available until March 31, 2021 for this study. All participants were followed from the date of visiting the assessment center until loss of follow-up, death, the first occurrence of diabetes mellitus, or end of this study (March 31, 2021), whichever came first.

### 2.5 Covariates

A range of covariates were considered in our statistical analysis, including: ethnicity (White, Asian, Black, Mixed, or Others), educational attainment (higher degree, vocational qualifications, any school degree, or other (Piernas et al., 2021)), household income, smoking status (never, previous, or current smoker), physical activity (assessed by a short-form International Physical Activity Questionnaire, which includes six questions of frequency and duration of walking, moderate-intensity and vigorous exercise: low, moderate, or high), and intake of vitamin supplements (yes or no, collected through the question “Do you regularly take any of the following?”). Since the supplement was also a source of vitamins that eventually entered the body to perform their biological activities, we included it as an additional covariate to adjust for. A cardiometabolic indicator, waist-to-hip ratio, were also considered as a measure of central obesity which was more commonly associated with diabetes mellitus (Smith, 2015).

### 2.6 Statistical analysis

To explore the associations of long-term exposure to air pollution and antioxidant vitamins intake with the incidence of diabetes mellitus, we constructed several Cox multivariable regression models. The first basic model incorporated socio-demographic factors only, including age, sex, ethnicity, education levels and household income. The second model further included lifestyle factors, including waist-to-hip ratio, physical activity, smoking status, alcohol intake, vitamin supplement intake, protein intake (Ancu et al., 2021, Tian et al., 2017), polyunsaturated fat (PUF) intake (Rice Bradley, 2018, Telle-Hansen et al., 2019), total sugar intake (Imamura et al., 2015, Tseng et al., 2021) and fiber intake (Reynolds et al., 2020, Weickert and Pfeiffer, 2018) in diet. In addition, we also fitted the shape of the exposure-response relationship between air pollution and diabetes mellitus using penalized spline models (Fig. S1).

We further conducted stratified analyses by dietary antioxidant vitamin intake (sufficient vs. insufficient) to examine its potential modifying effects on the associations between air pollution and diabetes mellitus by 2-sample z-tests using the stratification-specific point estimates and the SEs: $z=\left(\beta_{1}-\beta_{2}\right)/\sqrt{{\left({\mathrm{SE}}_{1}\right)}^{2}+{\left({\mathrm{SE}}_{2}\right)}^{2}}$; where *β*_*1*_ and *β*_*2*_ were the coefficients for a subgroup, and *SE*_*1*_ and *SE*_*2*_ were the respective standard errors (Altman and Bland, 2003).

### 2.7 Sensitivity analysis

Several sensitivity analyses were also applied to assess the results robustness. First, we additionally adjusted for Townsend deprivation index (a measure based on unemployment, non-car ownership, non-home ownership, and household overcrowding) (Tyrrell et al., 2016). This measure was used to control for the influence of area-level socioeconomic status. Second, we further included dietary antioxidant vitamins intake in the model to assess its impact on air pollution. Third, considering death as a competing risk event, we performed a sensitivity analysis using Fine-Gray subdistribution hazards regression model. Fourth, time-varying diet variables were incorporated to assess risk rather than a single time-fixed diet variable. Fifth, we excluded individuals reporting regular intake of vitamin supplements to minimize its influence.

All analyzes were conducted with R version 4.0.5, and a two-tailed *p* of < 0.05 indicated statistical significance.

## 3 Results

### 3.1 Baseline characteristics

The UK Biobank initially recruited 502,461 participants, among which, 345,971 participants were excluded for reasons discussed previously, with the remaining 156,490 included in this study (Fig. 1). The included participants were generally comparable with the overall population; for example, the distribution of socio-demographic and lifestyle characteristics and environmental exposures was similar (Table S1).

The mean age was 55.78 years [standard deviation (SD): 7.98]. About 54 % of the participants were women, and the majority were White (96 %). More than half of participants did not take any dietary supplement regularly. The average concentrations of PM_2.5_, PM_10_, NO_2_, and NOx were 9.90 (SD: 1.02) μg/m^3^, 19.37 (SD: 2.05) μg/m^3^, 29.38 (SD: 9.77) μg/m^3^, 43.08 (SD: 15.39) μg/m^3^, respectively (Table 1).

During a median follow-up of 11.7 years, 4271 incident diabetes mellitus cases were identified. Participants who developed diabetes mellitus were older, more likely to be males, had higher waist-to-hip ratio compared with those did not develop diabetes mellitus, and were more likely to be current or former smokers (Table 1).

**Fig. 2:**
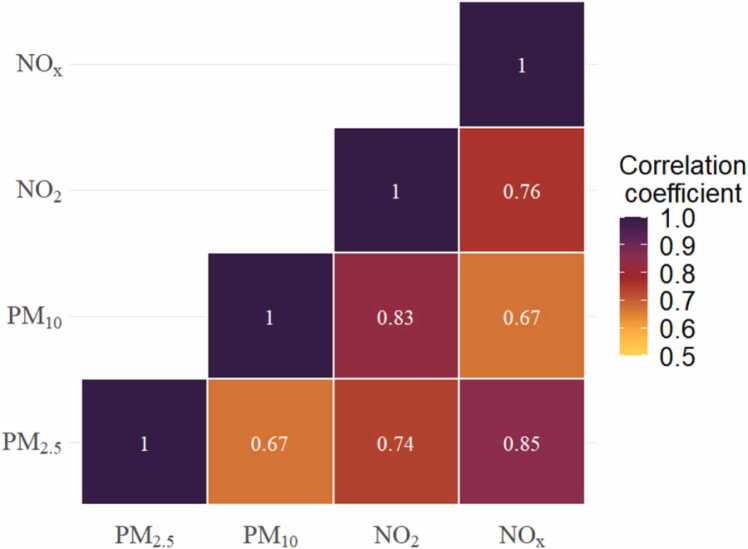
Correlation plot of air pollutants. PM_2.5_: is an abbreviation of fine particles, PM_10_: is an abbreviation of thoracic particles, NO_2_: is an abbreviation of nitrogen dioxide, NO_x_: is an abbreviation of nitrogen oxide.

Overall, the four pollutants were moderately-to-highly correlated. The correlation coefficients between PM_2.5_ and NO_x_ and that between PM_10_ and NO_2_ were much higher (correlation coefficient > 0.8) (Fig. 2).

### 3.2 Associations between air pollutants or antioxidant vitamins and diabetes

Significant positive associations were observed between exposure to all four air pollutants and incident diabetes mellitus (Table 2). Such associations were robust to adjustment for the wide array of covariates as consistent results were observed across different models. In fully-adjusted models, each 5 μg/m^3^ increase in PM_2.5_, PM_10_, NO_2_ and NO_x_ were associated with increased risks of diabetes mellitus, with hazard ratios (HRs) of 1.50 (95 % confidence interval (CI): 1.29, 1.73) for PM_2.5_, 1.15 (95 % CI: 1.07, 1.24) for PM_10_, 1.04 (95 % CI: 1.02, 1.05) for NO_2_ and 1.02 (95 % CI: 1.02, 1.03) for NO_x_ (Table 2).

**Table 2:** Association between exposure to air pollutants or antioxidant vitamins and the incidence of diabetes.

	Model 1^a^		Model 2^a^		Model 3^a^	
	HR	95% CI	HR	95 % CI	HR	95% CI
Sufficient antioxidant	0.88*	0.83,0.94	0.91*	0.85, 0.97	0.93*	0.86, 1.00
PM_2.5_ (5 μg/m^3^)^b^	1.64*	1.43,1.89	1.58*	1.37, 1.83	1.50*	1.29, 1.73
PM_10_ (5 μg/m^3^)^b^	1.17*	1.09,1.26	1.17*	1.09, 1.26	1.15*	1.07, 1.24
NO_2_ (5 μg/m^3^)^b^	1.04*	1.02,1.05	1.04*	1.03, 1.06	1.04*	1.02, 1.05
NO_x_ (5 μg/m^3^)^b^	1.03*	1.02,1.04	1.03*	1.02, 1.04	1.02*	1.02, 1.03

* *P* < 0.05.

a Model 1: is the basic model; Model 2: builds on Model 1, adjusting for demographic factors such as sex, age, ethnicity, education attainment and average household income;Model 3: builds on Model 2, adjusting for lifestyle factors such as waist to hip ratio, physical activity, smoking status, alcohol intake, vitamin supplement, protein intake, polyunsaturated fat intake, total sugar intake and fiber intake in diet.

b PM_2.5_ is an abbreviation of fine particles, PM_10_ is an abbreviation of thoracic particles, NO_2_ is an abbreviation of nitrogen dioxide, NO_x_ is an abbreviation of nitrogen oxide.

Similarly, sufficient intake of antioxidant vitamins was associated with a significant reduction in the incidence of diabetes mellitus after adjustment for covariates, with a HR of 0.93 (95 % CI: 0.86, 1.00) (*p* < 0.05) (Table 2).

### 3.3 Effect modification by vitamins intake

Compared to individuals with insufficient intake of antioxidant vitamins, those with sufficient intake had a weaker association between PM_2.5_ and the risk of diabetes mellitus [sufficient vs. insufficient: HR = 1.12 (95 % CI: 0.87, 1.45) vs. 1.69 (95 % CI: 1.42, 2.02)] (*p* for comparison = 0.01) (Table 3). Similar patterns were found for other air pollutants, except that the between-group differences for NO_x_ were statistically significant.

**Table 3:** Association between air pollutants and the incidence of diabetes stratified by the intake of antioxidant vitamins.^a^

Air pollutants (5 μg/m^3^)		Antioxidant vitamins		Vitamin A		Vitamin C		Vitamin E	
		Sufficient	Insufficient	Sufficient	Insufficient	Sufficient	Insufficient	Sufficient	Insufficient
PM_2.5_^b^	HR	1.12	1.69*	1.18	1.53*	1.35*	2.45*	1.09	1.60*
	95 % CI	0.87, 1.45	1.42, 2.02	0.89, 1.57	1.29, 1.81	1.16, 1.58	1.57, 3.84	0.76, 1.54	1.36, 1.87
	*p*^c^	0.01		0.13		0.01		0.05	
PM_10_^b^	HR	1.00	1.21*	1.06	1.13*	1.06	1.50*	0.99	1.18*
	95 % CI	0.88, 1.14	1.10, 1.34	0.91, 1.22	1.04, 1.24	0.98, 1.15	1.18, 1.91	0.82, 1.19	1.09, 1.29
	*p*^c^	0.02		0.42		0.01		0.08	
NO_2_^b^	HR	1.01	1.05*	1.01	1.04*	1.03*	1.10*	1.02	1.04*
	95 % CI	0.98, 1.04	1.03, 1.07	0.98, 1.04	1.02, 1.06	1.01, 1.04	1.05, 1.16	0.98, 1.06	1.02, 1.06
	*p*^c^	0.02		0.14		0.01		0.39	
NO_x_^b^	HR	1.01	1.03*	1.01	1.02*	1.02*	1.04*	1.00	1.03*
	95 % CI	0.99, 1.03	1.02, 1.04	1.00, 1.03	1.01, 1.04	1.01, 1.03	1.01, 1.07	0.98, 1.03	1.02, 1.04
	*p*^c^	0.06		0.32		0.25		0.05	

* *P* < 0.05 (*p* value for hazard ratio of association between air pollutants and diabetes mellitus incidence.

a Fully adjusted model: adjusting for sex, age, ethnicity, education attainment, average household income, waist to hip ratio, physical activity, smoking status, alcohol intake, vitamin supplement, protein intake, polyunsaturated fat intake, total sugar intake and fiber intake in diet.

b PM_2.5_ is an abbreviation of fine particles, PM_10_ is an abbreviation of thoracic particles, NO_2_ is an abbreviation of nitrogen dioxide, NO_x_ is an abbreviation of nitrogen oxide.

c *P* value for difference between the group of sufficient vitamin intake and the group of insufficient vitamin intake, is given by 2-sample z-test: $z=\left(\beta_{1}-\beta_{2}\right)/\sqrt{{\left({\mathrm{SE}}_{1}\right)}^{2}+{\left({\mathrm{SE}}_{2}\right)}^{2}}$, where *β*_*1*_ and *β*_*2*_ were the coefficients for a subgroup.

Consistently, when further dividing the overall intake into individual vitamins, we observed significant modifying effects by vitamin C or E for some air pollutants. For instance, the HR of developing diabetes mellitus per 5 μg/m^3^ increase in PM_2.5_ was 1.35 (95 % CI: 1.16, 1.58) and 2.45 (95 % CI: 1.57, 3.84) in participants with and without sufficient vitamin C intake respectively, and the HR was 1.09 (95 % CI: 0.76, 1.54) and 1.60 (95 % CI: 1.36, 1.87) in participants with and without sufficient vitamin E intake respectively (all *p* for comparison < 0.05).

In sensitivity analyses, additional adjustment for Townsend deprivation index or dietary antioxidant vitamin intakes did not alter the results materially. It also remained robust that the associations between long-term air pollution exposure and incident diabetes mellitus in the competing risk model and in the model that included effects of changes in dietary variables over time. Similar results were also found after excluding participants with regular intake of vitamin supplements (Tables S2–S3). In the cohort having irregular intake of vitamin supplements, modification by the dietary intake of antioxidant vitamins was significant for the relationships between PM_2.5_ and the incidence of diabetes mellitus, so as vitamin C or E.

## 4 Discussion

Our study revealed that long-term air pollution exposure was associated with an increased risk of diabetes mellitus. The associations were attenuated in participants who consumed sufficient antioxidant vitamins compared to those with insufficient intake. Similar results were found for each individual vitamin (i.e., vitamins C and E). The results provide the probability to explore the underlying mechanism of PM-related diabetes mellitus and to develop new individual-level interventions.

### 4.1 Comparison with other studies

There has been mounting evidence for the association between air pollution and diabetes mellitus incidence, and the positive associations observed in our study are consistent with most previous studies. For instance, one meta-analysis of 17 studies synthesized the evidence of the effect of ambient air pollutants on diabetes mellitus and showed that both gaseous pollutants and PM were associated with increased risks of diabetes-related morbidity, and the associations were stronger for gaseous pollutants than for PM (Janghorbani et al., 2014). Other recent meta-analyses (He et al., 2017, Ritz et al., 2019) also implied that long-term exposure to PM_2.5_ was significantly associated with an increased incidence of diabetes mellitus, with risk raised by 25 % (95 % CI: 10, 43) per 10 µg/m^3^ PM_2.5_. Two recent cohort studies conducted in Canada (Bai et al., 2018, Paul et al., 2020) demonstrated that traffic-related air pollution including NO_2_ may increase the diabetes mellitus risks. The results of our study add to the scientific evidence that long-term exposure to PM and NOx increases the risk of developing diabetes mellitus.

### 4.2 Potential mechanism

More recently, growing evidence based on experimental studies supports that oxidative stress is served as potential biological mechanism of how air pollution may induce diabetes mellitus. Both the heavy metal and organic compound components of atmospheric particulate matter and gaseous pollutants can react with endogenous substances directly to produce reactive oxygen species (ROS) or reactive nitrogen species (RNS) to induce oxidative stress (Chatkin et al., 2022, Glencross et al., 2020). These increased free radicals can cause the destruction of the mitochondrial structure in pancreatic beta cells, resulting in beta cell dysfunction (Li et al., 2019). ROS can also interfere with the insulin signaling pathway by activating the nuclear factor kappa beta signaling pathway to trigger a pro-inflammatory cascade, resulting in dysregulation of glucose and indirect inhibition of beta cell function (Li et al., 2019, Zhang et al., 2020). Furthermore, ROS and RNS may indirectly activate a number of cellular stress-sensitive pathways and then damage the tissues (Li et al., 2019). Moreover, the free radicals appear to depress the expression of some specific biological macromolecules, resulting in reduced insulin action, which may lead to vascular and even systemic insulin resistance (Li et al., 2019, Zhang et al., 2020). This series of adverse health events is an important mechanism of the occurrence and development of air-pollution-induced diabetes mellitus.

Given the significant role of oxidative stress played in air-pollution-induced diabetes, particularly PM, it is logical to posit that antioxidant vitamins have the potential to alleviate PM-associated diabetes mellitus. Vitamin A, C and E, as antioxidants, could reduce oxidative stress and inflammation through reacting with ROS induced by air pollution exposure (Ghezzi, 2020), thereby protecting metabolic function and reducing the possibility of developing diabetes mellitus. Furthermore, vitamin E could remove ROS from the body through the enzyme dependent antioxidant system in the body. For example, one study shows that vitamin E increases the expression of both nuclear factor-like 2 and Glutathione S-transferase in addition to reducing the expression of angiogenic matrix metalloproteinase-1 (Siti et al., 2015). The beneficial action of vitamin C in reducing hyperglycemia in those with diabetes mellitus is supported by a recent study (Mason et al., 2019), which reported that the antioxidant action of vitamin C was through a decrease in the plasma F_2_-isoprostanes. Carotenoids, the main source of vitamin A, can quench free radicals, prevent lipid oxidation, and activate the antioxidant cascade, thereby antagonizing the body's oxidative stress (Bohn et al., 2021).

### 4.3 Novel findings on antioxidant vitamins, air pollution and diabetes

In our study, we observed stronger associations between air pollution and diabetes mellitus among individuals with insufficient antioxidant vitamins intake compared with those with sufficient intake, suggesting that antioxidant intake may play a role in modifying the effect of air pollutions. This phenomenon has also been observed in previous investigations of the health effects of tobacco smoking (which also increase oxidative stress) on colorectal cancer, lung function decline, and asthma (Lee et al., 2015). To our knowledge, no study has so far examined the potential effect modification by antioxidant vitamins (vitamin A, C, and E) on the associations between ambient air pollutants and diabetes mellitus, and our study adds new knowledge on this topic.

Sufficient dietary intake of antioxidant vitamins is beneficial to human health. The human body is exposed to the environment every day and is affected by various potential exogenous risk factors, especially PM_2.5_, PM_10_, NO_2_, NO_x_ and other well-recognized risk factors, which induce the body's oxidative stress response and inflammation, thereby leading to an increased risk of diabetes mellitus and even other metabolic disorders. Antioxidants can antagonize the body's oxidative stress response, play a modification role, and reduce the risk of disease. The antioxidant defense system in the body is mainly synthesized by itself and generally cannot be supplemented from exogenous intake, while vitamin A, C and E can be supplemented from outside the body. Daily consumption of foods rich in vitamin A, C and E, such as vegetables and fruits, in accordance with the national dietary intake standards can help reduce the oxidative stress caused by exposure to exogenous air pollutants in the body, thereby protecting health. It also provides guidance for public health policy makers, such as governments, to encourage residents in areas with air pollution to consume enough fruits or vegetables and other foods rich in antioxidant vitamins at each meal.

### 4.4 Strengths of the study

This study had some important advantages. First, the prospective cohort study design and the large sample size along with the extensive information on various covariates enabled us to characterize the associations between air pollution and diabetes mellitus more accurately. Second, this study provided strong evidence for the modification of the effect of dietary antioxidant vitamins on the association between air pollution and diabetes mellitus.

### 4.5 Limitations of the study

Several limitations should also be acknowledged. First, the participants in the UK Biobank were not representative of the general population in the UK because they were recruited on a voluntary basis, and “healthy volunteer” selection bias was inevitable (Fry et al., 2017). Second, for the LUR models used for exposure assessment, only a restricted number of 20 monitoring sites were available for model development, which may have limited the predictive ability of the models (Eeftens et al., 2012). Third, concentrations of air pollutants and other covariates were only measured at baseline, which may have resulted in misclassification. Fourth, the diet of participants may change in 2013–2019, which may exist information bias. Finally, questionnaire-measured intake does not necessarily represent the amounts of vitamin that really exert antioxidant effects after exogenous intake to enter the human body through digestion, absorption and transformation. Other factors need to be taken into consideration, for example, that various substances have different conversion rates for diverse vitamins.

## 5 Conclusion

Our findings suggest that long-term exposure to air pollutants (PM_2.5_, PM_10_, NO_2_ and NO_x_) could increase the risk of diabetes mellitus, and sufficient dietary intake of antioxidants (such as vitamin A, C and E) may attenuate the adverse effects of air pollution.

## CRediT authorship contribution statement

**Haopeng Li:** Conceptualization, Methodology, Formal analysis, Data curation, Writing – original draft, Visualization. **Miao Cai:** Formal analysis. **Haitao Li:** Resources. **Zhengmin (Min) Qian:** Writing – review & editing. **Katie Stamatakis:** Writing – review & editing. **Stephen Edward McMillin:** Writing – review & editing. **Zilong Zhang:** Writing – review & editing. **Qiansheng Hu:** Writing – review & editing. **Hualiang Lin:** Resources, Writing – review & editing.

## Funding

This work was supported, in whole or in part, by the 10.13039/100000865Bill & Melinda Gates Foundation [Grant no.: INV-016826]. Under the Grant conditions of the Bill & Melinda Gates Foundation, a Creative Commons Attribution 4.0 Generic License has already been assigned to the Author Accepted Manuscript version that might arise from this submission.

## Declaration of Competing Interest

The authors declare that they have no known competing financial interests or personal relationships that could have appeared to influence the work reported in this paper.

## Data Availability

The authors do not have permission to share data.
