# Supplementary material for: Is dietary intake of antioxidant vitamins associated with reduced adverse effects of air pollution on diabetes? Findings from a large cohort study
Source: Ecotoxicol Environ Saf. 2022 Nov;246:114182. doi: 10.1016/j.ecoenv.2022.114182 (PMC9626446; doi:10.1016/j.ecoenv.2022.114182)
Supplement: Supplementary file 1 — Supplementary material [file mmc1.docx]

Table S1. The comparison of the basic characteristics of the included and excluded objects

| Variables | Overall (*n*=502,461) | Exclusion (*n*=345,971) | Inclusion (*n*=156,490) |
| --- | --- | --- | --- |
| Sex, *n* (%) |  |  |  |
| Female | 273,353 (54.40) | 188,420 (54.46) | 84,933 (54.27) |
| Male | 229,107 (45.60) | 157,550 (45.54) | 71,557 (45.73) |
| Age, mean (SD), years | 56.53 (8.10) | 56.87 (8.12) | 55.78 (7.98) |
| Ethnicity, *n* (%) |  |  |  |
| White | 472,657 (94.59) | 322,376 (93.93) | 150,281 (96.03) |
| Asian | 11,453 (2.29) | 9,045 (2.64) | 2,408 (1.54) |
| Black | 8,060 (1.61) | 6,278 (1.83) | 1,782 (1.14) |
| Mixed | 2,956 (0.59) | 1,998 (0.58) | 958 (0.61) |
| Others ^a^ | 4,558 (0.91) | 3,497 (1.02) | 1,061 (0.68) |
| Education attainment, *n* (%) |  |  |  |
| Higher degree | 233,570 (47.44) | 139,984 (41.68) | 93,586 (59.80) |
| Any school degree | 144,085 (29.27) | 99,871 (29.74) | 44,214 (28.25) |
| Vocational qualifications | 29,410 (5.97) | 22,140 (6.59) | 7,270 (4.65) |
| Other | 85,265 (17.32) | 73,845 (21.99) | 11,420 (7.30) |
| Household income, *n* (%) |  |  |  |
| Less than 18,000 | 97,192 (19.58) | 76,574 (22.52) | 20,618 (13.18) |
| 18,000 to 30,999 | 108,172 (21.79) | 74,340 (21.87) | 33,832 (21.62) |
| 31,000 to 51,999 | 110,762 (22.31) | 69,529 (20.45) | 41,233 (26.35) |
| 52,000 to 100,000 | 86,256 (17.37) | 49,666 (14.61) | 36,590 (23.38) |
| Greater than 100,000 | 22,928 (4.62) | 11,700 (3.44) | 11,228 (7.17) |
| Unknown | 71,139 (14.33) | 58,150 (17.11) | 12,989 (8.30) |
| Waist to hip ratio, mean (SD) | 8.72 (0.90) | 8.76 (0.90) | 8.62 (0.88) |
| Smoking status, *n* (%) |  |  |  |
| Never | 273,497 (54.75) | 184,358 (53.75) | 89,139 (56.96) |
| Previous | 173,044 (34.64) | 117,660 (34.30) | 55,384 (35.39) |
| Current | 52,971 (10.60) | 41,004 (11.95) | 11,967 (7.65) |
| Physical activity, *n* (%) |  |  |  |
| Low | 76,206 (18.94) | 48,013 (19.53) | 28,193 (18.02) |
| Moderate | 164,012 (40.76) | 97,867 (39.81) | 66,145 (42.27) |
| High | 162,131 (40.30) | 99,979 (40.67) | 62,152 (39.72) |
| Vitamin supplement, *n* (%) |  |  |  |
| No | 359,946 (72.01) | 249,509 (72.67) | 110,437 (70.57) |
| Yes | 139,882 (27.99) | 93,829 (27.33) | 46,053 (29.43) |
| PM_2.5_, mean (SD), μg/m^3 b^ | 9.99 (1.06) | 10.04 (1.07) | 9.90 (1.02) |
| PM_10_, mean (SD), μg/m^3 b^ | 19.23 (1.94) | 19.17 (1.88) | 19.37 (2.05) |
| NO_2_, mean (SD), μg/m^3 b^ | 29.07 (9.06) | 28.93 (8.71) | 29.38 (9.77) |
| NO_x_, mean (SD), μg/m^3 b^ | 44.11 (15.53) | 44.59 (15.57) | 43.08 (15.39) |

^a^ "Others" means any ethnicity other than White, Black, Asian, or Mixed ethnicity.

^b^ PM_2.5_: is an abbreviation of fine particles, PM_10_: is an abbreviation of thoracic particles, NO_2_: is an abbreviation of nitrogen dioxide, NO_x_: is an abbreviation of nitrogen oxide.

Table S2. Association between exposure to air pollutants and the incidence of diabetes

| Air pollutants  (5μg/m^3^) | Model 4^a^ | | Model 5^a^ | | Model 6^a^ | | Model 7^a^ | | Model 8^ab^ | |
| --- | --- | --- | --- | --- | --- | --- | --- | --- | --- | --- |
|  | HR | 95% CI | HR | 95% CI | HR | 95% CI | HR | 95% CI | HR | 95% CI |
| Sufficient antioxidant | 0.93^*^ | 0.86, 1.00 | 0.93^*^ | 0.86, 1.00 | 0.92^*^ | 0.86, 0.99 | 0.88^*^ | 0.82, 0.95 | 0.94 | 0.86，1.03 |
|  |  |  |  |  |  |  |  |  |  |  |
| PM_2.5_ ^c^ | 1.23^*^ | 1.05, 1.44 | 1.50^*^ | 1.29, 1.73 | 1.59^*^ | 1.38, 1.83 | 1.51^*^ | 1.30, 1.75 | 1.57^*^ | 1.32, 1.87 |
| PM_10_ ^c^ | 1.02 | 0.94, 1.11 | 1.15^*^ | 1.07, 1.24 | 1.20^*^ | 1.11, 1.30 | 1.17^*^ | 1.08, 1.27 | 1.21^*^ | 1.10, 1.32 |
| NO_2_ ^c^ | 1.01 | 0.99, 1.03 | 1.04^*^ | 1.02, 1.05 | 1.05^*^ | 1.03, 1.06 | 1.04^*^ | 1.02, 1.06 | 1.05^*^ | 1.03, 1.07 |
| NO_x_ ^c^ | 1.01^*^ | 1.00, 1.02 | 1.02^*^ | 1.02, 1.03 | 1.03^*^ | 1.02, 1.04 | 1.02^*^ | 1.02, 1.03 | 1.03^*^ | 1.02, 1.04 |

^a^ Model 4: adjusting for sex, age, ethnicity, education attainment, average household income, waist to hip ratio, physical activity, smoking status, alcohol intake, vitamin supplement, protein intake, polyunsaturated fat intake, total sugar intake and fiber intake in diet, and deprive index, additionally;

Model 5: adjusting for sex, age, ethnicity, education attainment, average household income, waist to hip ratio, physical activity, smoking status, alcohol intake, vitamin supplement, protein intake, polyunsaturated fat intake, total sugar intake and fiber intake in diet, and antioxidant vitamins intake, additionally;

Model 6: competing risk model adjusting for sex, age, ethnicity, education attainment, average household income, waist to hip ratio, physical activity, smoking status, alcohol intake, vitamin supplement, protein intake, polyunsaturated fat intake, total sugar intake and fiber intake in diet;

Model 7: All diet variables as time-varying variables and the model adjusting for sex, age, ethnicity, education attainment, average household income, waist to hip ratio, physical activity, smoking status, alcohol intake, vitamin supplement, protein intake, polyunsaturated fat intake, total sugar intake and fiber intake in diet;

Model 8: adjusting for sex, age, ethnicity, education attainment, average household income, waist to hip ratio, physical activity, smoking status, alcohol intake, protein intake, polyunsaturated fat intake, total sugar intake and fiber intake in diet.

^b^ People who answered at baseline that they regularly took vitamin supplements were excluded.

^c^ PM_2.5_ is an abbreviation of fine particles, PM_10_ is an abbreviation of thoracic particles, NO_2_ is an abbreviation of nitrogen dioxide, NO_x_ is an abbreviation of nitrogen oxide.

^*^ *P* < 0.05

Table S3. Association between air pollutants and the incidence of diabetes modified by the intake of antioxidant vitamins ^ab^

| Air pollutants  (5μg/m^3^) |  | Antioxidant vitamins | | Vitamin A | | Vitamin C | | Vitamin E | |
| --- | --- | --- | --- | --- | --- | --- | --- | --- | --- |
|  |  | Sufficient | Insufficient | Sufficient | Insufficient | Sufficient | Insufficient | Sufficient | Insufficient |
| PM_2.5_ ^c^ | HR | 1.14 | 1.78^*^ | 1.29 | 1.56^*^ | 1.41^*^ | 2.57^*^ | 1.03 | 1.70^*^ |
|  | 95% CI | 0.84, 1.57 | 1.45, 2.19 | 0.92, 1.81 | 1.28, 1.90 | 1.17, 1.69 | 1.53, 4.30 | 0.66, 1.59 | 1.41, 2.05 |
|  | *p*^d^ | 0.02 | | 0.34 | | 0.03 | | 0.04 | |
| PM_10_ ^c^ | HR | 1.05 | 1.26^*^ | 1.13 | 1.17^*^ | 1.12^*^ | 1.45^*^ | 0.98 | 1.25^*^ |
|  | 95% CI | 0.90, 1.24 | 1.13, 1.41 | 0.95, 1.35 | 1.05, 1.30 | 1.01, 1.23 | 1.10, 1.93 | 0.78, 1.22 | 1.13, 1.38 |
|  | *p*^d^ | 0.07 | | 0.73 | | 0.09 | | 0.05 | |
| NO_2_ ^c^ | HR | 1.02 | 1.06^*^ | 1.02 | 1.05^*^ | 1.03^*^ | 1.10^*^ | 1.01 | 1.05^*^ |
|  | 95% CI | 0.98, 1.05 | 1.03, 1.08 | 0.99, 1.06 | 1.02, 1.07 | 1.01, 1.06 | 1.04, 1.16 | 0.97, 1.06 | 1.03, 1.08 |
|  | *p*^d^ | 0.05 | | 0.32 | | 0.06 | | 0.15 | |
| NO_x_ ^c^ | HR | 1.01 | 1.03^*^ | 1.02 | 1.03^*^ | 1.03^*^ | 1.04^*^ | 1.00 | 1.03^*^ |
|  | 95% CI | 0.99, 1.03 | 1.02, 1.05 | 1.00, 1.04 | 1.02, 1.04 | 1.01, 1.04 | 1.01, 1.07 | 0.97, 1.03 | 1.02, 1.05 |
|  | *p*^d^ | 0.06 | | 0.51 | | 0.50 | | 0.02 | |

^a^ Fully adjusted model: adjusting for sex, age, ethnicity, education attainment, average household income, waist to hip ratio, physical activity, smoking status, alcohol intake, vitamin supplement, protein intake, polyunsaturated fat intake, total sugar intake and fiber intake in diet.

^b^ People who answered at baseline that they regularly took vitamin supplements were excluded.

^c^ PM_2.5_ is an abbreviation of fine particles, PM_10_ is an abbreviation of thoracic particles, NO_2_ is an abbreviation of nitrogen dioxide, NO_x_ is an abbreviation of nitrogen oxide.

^d^ *P* value for difference between the group of sufficient vitamin intake and the group of insufficient vitamin intake, is given by 2-sample z-test: $z= \left( \beta_{1}-\beta_{2} \right)/\sqrt{\left( {SE}_{1} \right)^{2}+\left( {SE}_{2} \right)^{2}}$, where *β_1_* and *β_2_* were the coefficients for a subgroup.

^*^ *P* < 0.05 (*p* value for hazard ratio of association between air pollutants and diabetes mellitus incidence)


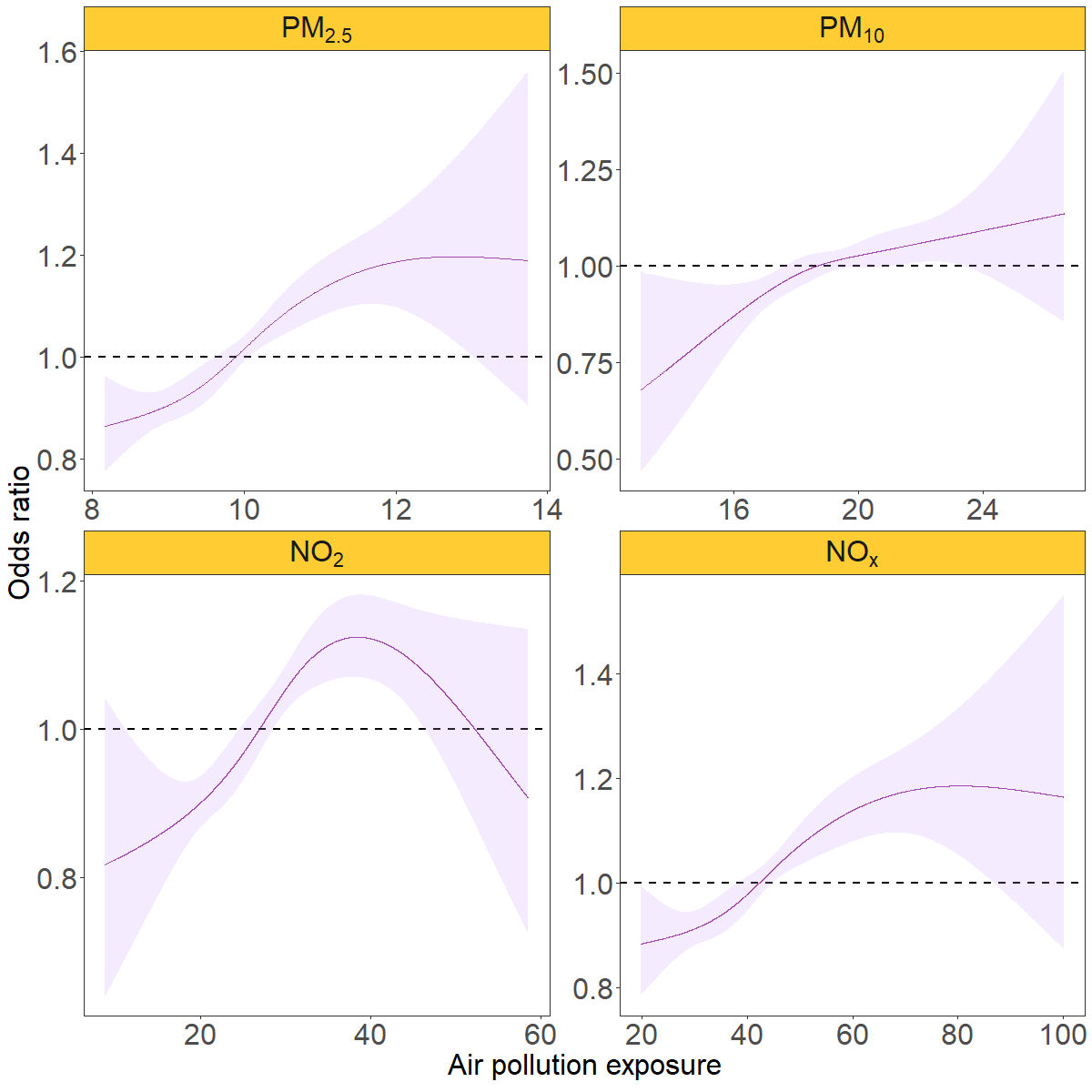


Fig. S1. The dose-response curve of the association of diabetes risk with ambient PM_2.5_, PM_10_, NO_2_ and NO_x_.

PM_2.5_ is an abbreviation of fine particles, PM_10_ is an abbreviation of thoracic particles, NO_2_ is an abbreviation of nitrogen dioxide, NO_x_ is an abbreviation of nitrogen oxide. The line represents the estimated odds ratio of diabetes mellitus risk, and shaded areas represent 95% CI. The models were adjusted for sex, age, ethnicity, education attainment, average household income, waist to hip ratio, physical activity, smoking status, alcohol intake, vitamin supplement intake, protein intake, polyunsaturated fat intake, total sugar intake and fiber intake in diet.
